# Prime editing using paired pegRNAs targeting NG- or NGG-PAM in rice

**DOI:** 10.3389/fgeed.2025.1550308

**Published:** 2025-08-28

**Authors:** Ayako Nishizawa-Yokoi, Keiko Iida, Akiko Mori, Seiichi Toki

**Affiliations:** ^1^ Institute of Agrobiological Sciences, National Agriculture and Food Research Organization (NARO), Tsukuba, Japan; ^2^ Graduate School of Nanobioscience, Yokohama City University, Yokohama, Kanagawa, Japan; ^3^ Faculty of Agriculture, Ryukoku University, Otsu, Shiga, Japan

**Keywords:** prime editing, paired pegRNAs, PAM, SpCas9-NG, rice

## Abstract

Prime editing (PE) enables precise genome modification, i.e., all 12 types of base substitution, as well as designed insertion and deletion. Previously, we developed an efficient PE system using a pair of engineered pegRNAs (epegRNAs), appending an RNA pseudoknot sequence to the 3′ends of pegRNAs to enhance stability and prevent degradation of the 3′extension. Using a wild-type nSpCas9-based PE system (PE-wt) recognizing an NGG-protospacer adjacent motif (PAM) in this approach, two NGG-PAMs (NGG and CCN) adjacent to the target site are required for targeting by paired pegRNAs; however, this is not the PAM configuration available at most target sites. Using an nSpCas9-NG variant recognizing NG-PAM in PE (PE-NG) can expand applicability. Here, we compare the PE efficiency of PE-wt with paired epegRNAs targeting a distal NGG-PAM *versus* PE-NG with paired epegRNAs targeting NG-PAMs adjacent to the target site. By introducing substitution and designated deletion mutations into target genes via PE-wt and PE-NG with paired epegRNAs, we demonstrated that PE-wt could edit the target site efficiently despite targeting the distal PAM site when either of the paired epegRNAs for PE-NG targets PGC-PAM. If epegRNAs for PE-NG are designed to recognize NGA and NGT-PAM, there is no significant difference in frequency between PE-NG and PE-wt. These findings indicate that PE efficiency via PE-NG is particularly low at the NGC-PAM in rice.

## 1 Introduction

Prime editing (PE) allows the introduction of desired mutations, i.e., all 12 types of base substitution, designed insertion, and designed deletion, using the combination of a fusion protein [nickase form of Cas9 protein (nCas9) + Moloney murine leukemia virus reverse transcriptase (RT)] and a PE guide RNA (pegRNA) composed of a single guide RNA and 3′extension containing the RT template and primer-binding site (Anzalone et al., 2019). Since it was first reported, many research groups have put great effort into developing efficient and reproducible PE systems (Chen and Liu, 2023). We have developed an efficient PE system (Nishizawa-Yokoi et al., submitted) through combining a paired pegRNAs approach (Lin et al., 2021) with an engineered pegRNA (epegRNA) with an RNA pseudoknot sequence added to the 3′ends of the pegRNAs (Nelson et al., 2022). Applying a canonical *Streptococcus pyogenes* Cas9 (SpCas9) nickase-based PE system (PE-wt) recognizing NGG-PAM to this approach, two NGG-PAMs (NGG and CCN) adjacent to the target site are required for targeting by paired epegRNAs; however, this is not the PAM configuration available at most target sites. Lin et al. (2021) reported that dual-pegRNA could theoretically target only 21.5% of genomic bases by computational analysis of the rice reference genome [IRGSP-1.0, (Kawahara et al., 2013),] when the PE-target was defined as extending from +1 to +15 and the canonical SpCas9 was used. To overcome the target range limitations of SpCas9, engineered Cas9 variants, e.g., SpCas9-NG, that recognize NG PAMs (Nishimasu et al., 2018) and a near PAM-less SpCas9 [SpRY, (Walton et al., 2020),], have been developed and adapted to nuclease, base editor, and PE (Kweon et al., 2021; Nishimasu et al., 2018; Walton et al., 2020). Applying a SpCas9-NG to the dual-pegRNA strategy has been reported to target potentially 89.2% of genomic bases in the rice genome (Lin et al., 2021).

We have also reported the successful adoption of SpCas9-NG to the prime editor (PE-NG) for the precise modification of endogenous target genes in rice. Comparing PE frequency via a wild-type nSpCas9-based PE system (PE-wt, targeting NGG-PAM) with PE-NG at several targets, precise modifications via PE-wt with paired epegRNAs targeting distant NGG-PAM were found to be more frequent than that using PE-NG with paired epegRNAs targeting adjacent NG-PAM when either of the paired epegRNAs for PE-NG targeted PGC-PAM.

## 2 Methods

### 2.1 Vector construction

The paired epegRNAs and 8-nt linkers between PBS and tevopreQ1 for epegRNAs were designed with pegFinder (http://pegfinder.sidichenlab.org/) (Chow et al., 2021) or PlantPegDesinger (http://www.plantgenomeediting.net/) (Lin et al., 2021) and pegLIT (https://peglit.liugroup.us/) (Nelson et al., 2022), respectively. To construct the epegRNA expression cassettes, oligonucleotide pairs pegRNA-F/R, including a 20-nt complementary sequence at each 5′and 3′end, respectively, were synthesized. Fragments synthesized by overlap extension PCR (Nelson and Fitch, 2011) (the 20-nt pegRNA spacer scaffold sequenceRT templatePBS8-nt linker) were introduced into the *Bbs*I site of pOsU6_BbsIx2_tQ1_polyT (Nishizawa-Yokoi et al., manuscript submitted) using the In-fusion HD cloning system (Takara). Alternatively, complementary oligonucleotides, pegRNA_5′F/5′R and pegRNA_3′F_Lt/3′R_Lt (listed in Supplementary Table S1), which consist of the 20-nt pegRNA spacer5′-half of scaffold sequence and 3′-half of scaffold sequenceRT templatePBS8-nt linker, respectively, were annealed and ligated together into the *Bbs*I site of pOsU6_BbsIx2_tQ1_polyT.

The PE-NG expression vector, pZH/PE-NG (Os Opt), was constructed from the CRISPR/Cas9 expression vector described in a previous study as pPZP_ZmUbi:BlCas9 (Negishi et al., manuscript submitted). The BlCas9 coding sequence was digested with *Pme*I/*Kpn*I from the pPZP_ZmUbi:BlCas9 vector and replaced with an *Oryza sativa* codon-optimized SpCas9-NG (R1335V/L1111R/D1135V/G1218R/E1219F/A1322R/T1337R) coding sequence (Endo et al., 2019; Nishimasu et al., 2018) using a conventional ligation method. The SpCas9-NG expression vector was digested with *Aat*I/*BsrG*I, and an N863A mutation was introduced by inserting a SpCas9-N863A fragment amplified by PCR using the primer sets listed in Supplementary Table S1 using the In-fusion HD cloning system (Takara). An *O. sativa* codon-optimized M-MLV RT coding sequence [SV40 NLS_engineered M-MLV RT (D200N/L603W/T330P/T306K/W313F) (Anzalone et al., 2019)_SV40 NLS] was amplified by PCR and introduced into the *Kpn*I/*Sac*I site located at the C-terminal region of nSpCas9-NG N863A using the In-fusion HD cloning system (Takara). The paired epegRNA expression cassettes were amplified independently by PCR with the primers shown in Supplementary Table S1, and were introduced concomitantly into the *Asc*I/*Pac*I site of pZH/PEwt (Os Opt) (Nishizawa-Yokoi et al., manuscript submitted) or pZH/PE-NG vector using the In-Fusion Snap cloning kit (Takara). These binary vectors were transformed into *Agrobacterium tumefaciens* EHA105 (Hood et al., 1993) by electroporation (Bio-Rad).

### 2.2 Plant materials and transformation of rice callus

Rice (*O. sativa* L.) cv. Nipponbare or the high-yield cultivar Yamadawara were used for the editing the *OsEPSPS* or *OsHSL1* gene, respectively. Four-week-old rice calli derived from embryo scutellum were used for *Agrobacterium*-mediated transformation following the procedure of Toki et al. (2006). After 3 days co-cultivation, transformed calli were washed and transferred to N6D medium containing 50 mg/L hygromycin B (Wako Pure Chemicals) and 25 mg/L meropenem (Wako Pure Chemicals) for 2 weeks. Antibiotic-resistant clonal callus lines were transferred to new N6D medium containing 50 mg/L hygromycin B and 25 mg/L meropenem and cultured for a further week. Transgenic callus lines carrying the desired mutations in the target gene were transferred to regeneration medium containing 25 mg/L meropenem and cultured for 4 weeks for plant regeneration. T_1_ progeny plants were obtained from self-pollinating T_0_ plants containing desired mutations in the *OsEPSPS* locus and were subjected to genotyping of the *OsEPSPS* gene to confirm the existence of the transgene. T_2_ progeny plants were obtained from T_1_ plants carrying the homozygous *OsEPSPS-PS* gene and were subjected to a herbicide-susceptibility test.

### 2.3 Genomic DNA extraction and analysis of PE frequency

Genomic DNA was extracted from clonal propagated transgenic calli according to the protocol for rapid DNA extraction reported by Kasajima et al. (2004). PE frequency in transgenic rice calli was calculated as the ratio of the number of calli carrying the targeted mutation, as detected by direct sequencing of PCR products, to the total number of transgenic calli analyzed.

To detect the targeted PE-induced mutation in the transgenic calli, and to confirm the existence of the desired mutation and undesired scaffold-derived byproducts in regenerated plants, PCR amplification was performed with KOD one PCR master mix (TOYOBO) using the primer sets listed in Supplementary Table S2. The PCR products were purified with AMPure XP (Beckman Coulter) and subjected to sequencing analysis using a Sanger sequencing kit (Thermo Fisher).

### 2.4 Glyphosate-susceptibility test

Seeds of wild-type rice (Nipponbare) and T_2_ progeny plants carrying homozygous *OsEPSPS-PS* gene were sown on 1/2MS medium with or without glyphosate (NACALAI TESQUE, INC.) and grown in a growth chamber at 27°C under a 16 h photoperiod.

### 2.5 Statistical analysis

When comparing two data sets, Student’s t*-*test was used and significant differences are indicated with asterisks (*p < 0.05).

## 3 Results

### 3.1 Comparing PE frequency in transgenic callus between paired epegRNAs with PE-NG targeting adjacent NG-PAMs and PE-wt targeting distant NGG-PAMs

**FIGURE 1:**
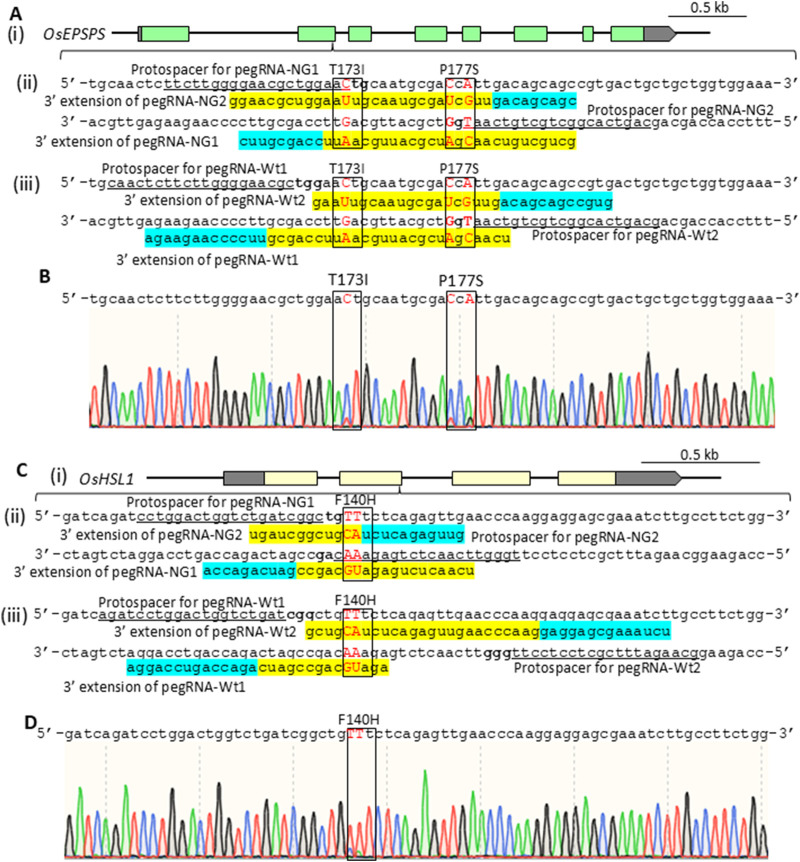
**(A)** Schematic of the OsEPSPS target site (i) and target sequence to introduce T173I (aCt to aTt) and P177S (CcA to TcG) mutations via PE‐NG (ii) or PE‐wt (iii). **(B)** Representative sequence chromatogram of PCR products spanning the target site in the OsEPSPS gene from transgenic calli. The positions of T173I and P177S mutations are boxed. **(C)** Schematic of the rice HPPD inhibitor sensitive‐like 1 (OsHSL1) gene target site (i) and target sequence to introduce an F140H (TTt to CAt) mutation via PE‐NG (ii) or PE‐wt (iii). **(D)** Representative sequence chromatogram of PCR products spanning the target site in the OsHSL1 gene from transgenic calli. The positions of the F140H mutations are boxed. The sequences shown below the target sequences reveal the 3° extension region of the epegRNAs. PBS and RT templates are highlighted in light blue and yellow, respectively. The protospacer sequences for pegRNAs are underlined. The positions of T173I and P177S mutations in OsEPSPS **(A)** or F140H mutation in OsHSL1 **(C)** are boxed. Target nucleotides are shown in red.

**FIGURE 2:**
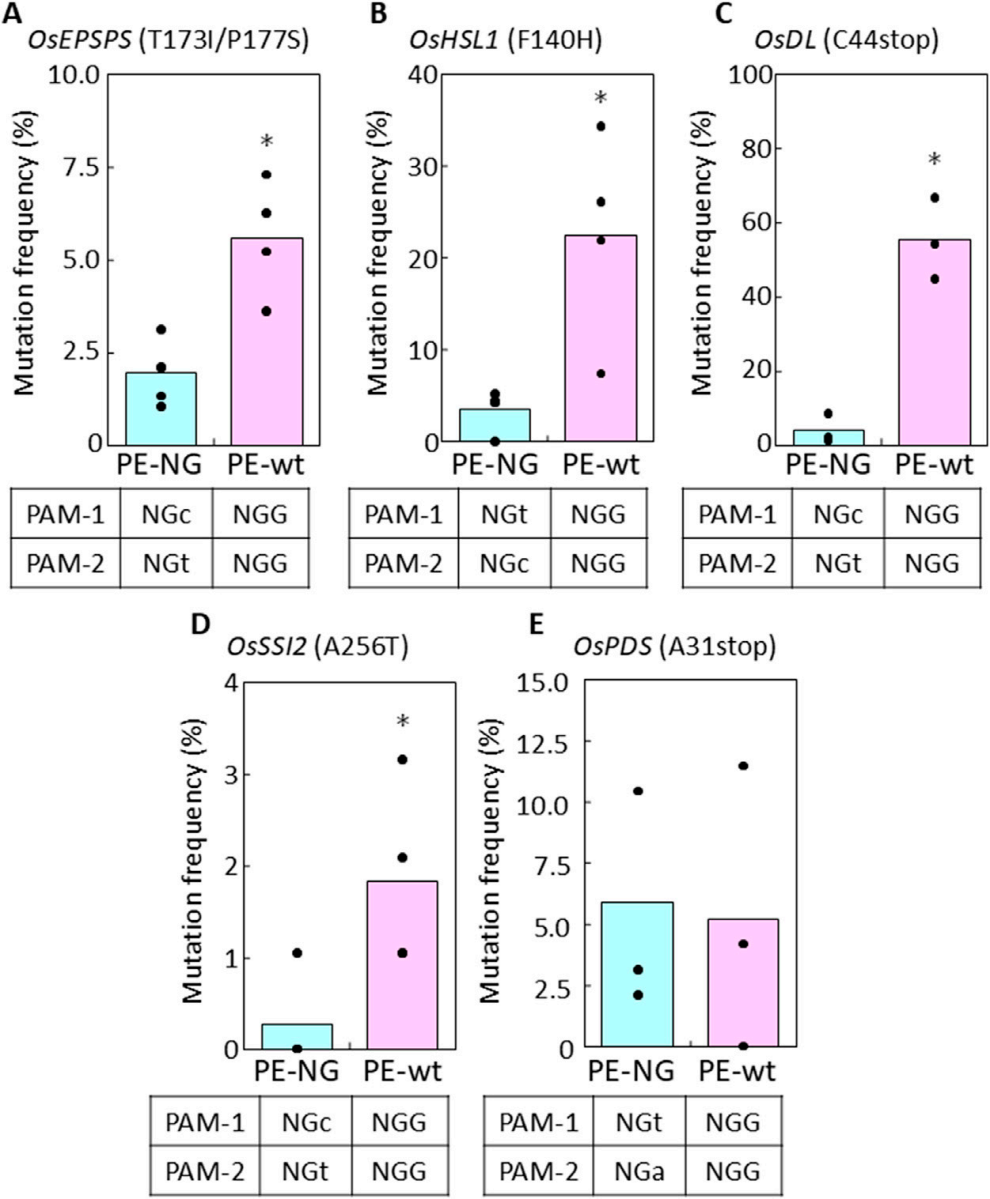
PE frequency in transgenic calli expressing PE-NG or PE-wt with paired epegRNAs. **(A–E)** PE frequency in transgenic calli expressing PE-NG and PE-wt with paired epegRNAs targeting *OsEPSPS* **(A)**, *OsHSL1* **(B)**, *OsDL* **(C)**, *OsSSI2* **(D)**, and *OsPDS* **(E).** A total of 96 independent transgenic callus lines were cloned and subjected to sequencing analysis to identify mutations in the target genes in each transformation experiment. More than three biological replicates were performed and calculated values are shown as dots on bar graphs indicating mean percentage scores. Differences were assessed by Student’s t-test (*p < 0.05).

To compare PE efficiency using PE-NG with an nSpCas9-NG variant targeting NG-PAM (Nishimasu et al., 2018) and PE-wt with a canonical nSpCas9 targeting NGG-PAM, we designed two pairs of epegRNAs introducing T173I and P177S (TIPS) mutations conferring resistance to the herbicide glyphosate into the rice five-enolpyruvylshikimate-3-phosphate synthase (*OsEPSPS*) gene (Achary et al., 2020) (Figure 1Ai). Using PE-NG, paired epegRNAs were designed to recognize NG-PAM (NG and CN) adjacent to the editing sites, resulting in nicking at a site adjacent to the target sites with the short available RT template (Figure 1Aii). On the other hand, we used paired epegRNAs targeting NGG-PAM (NGG and CCN) distal from the target site for PE-wt (Figure 1Aiii). A binary vector for PE-NG or PE-wt with paired epegRNAs was transformed into rice calli to test PE efficiency at the *OsEPSPS* locus. We detected TIPS mutations in *OsEPSPS* (*OsEPSPS-TIPS*) in PCR products spanning the target site generated from genomic DNA extracted from transgenic calli. Sequence analysis showed that TIPS mutations were found in 1.9% and 5.6% of transgenic calli carrying PE-NG and PE-wt with paired epegRNAs, respectively (Figures 1B, 2A). Next, we compared PE frequency when introducing the F140H (TTt to CAt) mutation, which confers tolerance to the HPPD herbicide, into the rice HPPD Inhibitor sensitive-like 1 (*OsHSL1*) gene (Figure 1Ci), between PE-NG and PE-wt (Dong et al., 2024) (Figure 1C(ii), (iii)). The desired modifications were detected by PCR/sequencing analysis in only a few percent and in >20% of callus lines transformed with paired epegRNAs and PE-NG or PE-wt, respectively (Figures 1D, 2B).

We also evaluated PE frequency between PE-NG and PE-wt with paired epegRNAs at another three target sites in rice calli (Supplementary Figures S1A-C). We designed paired epegRNAs for PE-NG and PE-wt to convert C44 to stop (-GC), A256 to T (G to A), and A31 to stop (G to T and -C) in the drooping leaf (*OsDL*) gene, the suppressor of salicylate insensitivity 2 (*OsSSI2*) gene, and the phytoene desaturase (*OsPDS*) gene, respectively (Supplementary Figures S1A-C). The paired epegRNAs for PE-NG recognized NGC-/NGT-, NGC-/NGT-, and NGT-/NGA-PAM in *OsDL*, *OsSSI2*, and *OsPDS*, respectively. *OsDL* and *OsPDS* have been used as selective marker genes for genome editing in several plant species (Lin et al., 2021; Mikami et al., 2015), because knockout of these genes has been reported to lead to albino (Qin et al., 2007) and drooping leaf (Yamaguchi et al., 2004) phenotypes, respectively. Substitution of A256T in *OsSSI2*—one of two amino acid mutations reported as a novel mutant allele of *Arabidopsis SSI2* (Yang et al., 2016)—could potentially confer disease resistance on rice. We found that PE-NG (3.82% at *OsDL* and 0.26% at *OsSSI2*) was less efficient than PE-wt at the *OsDL* and *OsSSI2* loci (55.21% at *OsDL* and 1.83% at *OsSSI2*, Figures 2C,D), while there was no significant difference in frequency between PE-NG and PE-wt at the *OsPDS* gene (5.90%, PE-NG and 5.21%, PE-wt; Figure 2E).

### 3.2 Mutation frequency in plants regenerated from transgenic callus expressing PE-NG or PE-wt

**FIGURE 3:**
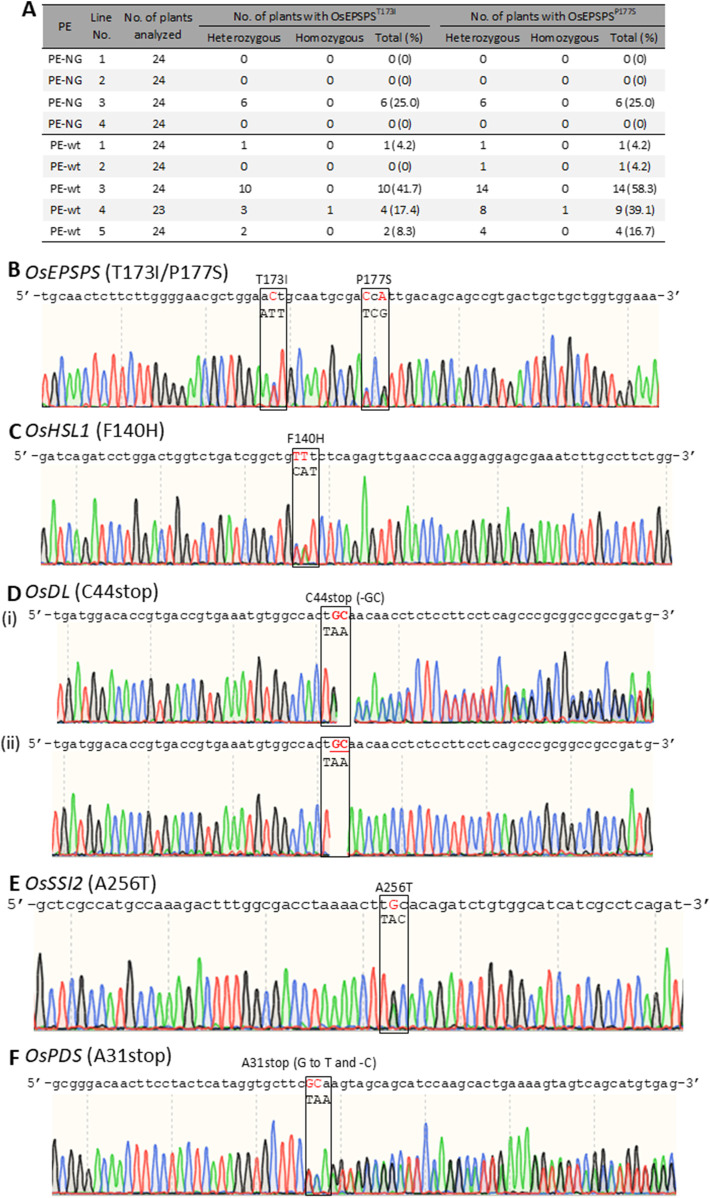
Sequence analysis of modified target genes via PE with paired epegRNAs in regenerated plants. A, Summary of genotyping of the *OsEPSPS* gene in regenerated plants expressing PE-NG or PE-wt with paired epegRNAs. B-F, Representative sequence chromatogram of PCR products spanning the target site in the *OsEPSPS* **(B)**, *OsHLS1* **(C)**, *OsDL* **(D)**, *OsSSI2* **(E)**, and *OsPDS* **(F)** gene from plants harboring a homozygous *OsEPSPS-PS* gene. Hetero (i) and homozygous (ii) T0 mutant plants were obtained from transgenic callus lines expressing PE-wt and epegRNAs targeting *OsDL* gene **(D)**. The positions of the target amino acid residues are boxed. Target nucleotides are shown in red.

Transgenic calli with *OsEPSPS-TIPS* were transferred to regeneration medium to obtain mutant plants harboring the desired mutations; 24 plants were obtained from each of four and five independent transgenic lines expressing PE-NG and PE-wt coupled with paired epegRNAs, respectively, and the genotypes of their *OsEPSPS* genes were determined by PCR and sequence analysis. Either T173I or P177S mutations (TIPS) or both were detected in at least one plant from all transgenic callus lines transformed with PE-wt (Figure 3A). On the other hand, we found *OsEPSPS-TIPS* mutant plants from only one line transformed with PE-NG (Figure 3A). In addition, no undesired by products were detected in any regenerated plants expressing either PE-NG or PE-wt (Figures 3A,B). Furthermore, transgenic plants were regenerated from callus lines carrying the PE-modified *OsHSL1, OsDL, OsSSI2, and OsPDS* genes. Between 7 and 24 plants were obtained from each transgenic callus line expressing PE-NG and PE-wt coupled with paired epegRNAs, and the genotypes of their target genes were determined by PCR and sequence analysis (Table 1; Figures 3C–F). Multiple plants harboring a heterozygous or homozygous F140H mutation in the *OsHSL1* gene were observed in all lines transformed with PE-wt (Table 1; Figure 3C). Similarly, in the *OsDL, OsSSI2, and OsPDS* genes, the proportion of regenerated plants carrying the hetero or homozygous desired mutation derived from callus lines expressing PE-wt was consistently higher than that of regenerated plants expressing PE-NG (Table 1; Figures 3D–F). However, undesired scaffold-derived byproducts in *OsHSL1*, *OsDL*, and *OsSSI2* were detected concomitantly in several regenerated plants (Table 1; Supplementary Figures S2A-C).

**TABLE 1:** Summary of genotyping of target genes in regenerated plants expressing PE-NG or PE-wt with paired epegRNAs cassettes**.**

Target gene	Mutation introduced by PE	PE	PAM sequences		Line no.	No. of T0 plants analyzed	No. of plants with only desired modification in target gene			No. of plants with undesired mutations in addition to desired modification		
			pegRNA-1	pegRNA-2			Heterozygous	Homozygous	Total (%)	InDel	Byproducts	Total (%)
OsHSL1	F140H	PE-NG	NGt	NGc	1	24	4	20	24 (100)	0	24	24 (100)
					2	24	0	0	0 (0)	0	0	0 (0)
					3	24	0	0	0 (0)	0	0	0 (0)
					4	24	0	24	24 (100)	0	0	0 (0)
					5	24	0	0	0 (0)	0	0	0 (0)
		PE-wt	NGG	NGG	1	24	20	2	22 (91.7)	0	7	7 (29.2)
					2	23	4	0	4 (17.4)	0	2	2 (8.7)
					3	24	3	0	3 (12.5)	0	0	0 (0)
					4	24	2	0	2 (8.3)	0	0	0 (0)
					5	24	16	0	16 (66.7)	3	12	15 (62.5)
OsDL	C44_stop	PE-NG	NGc	NGt	1	24	1	0	1 (4.2)	0	0	0 (0)
	(-GC)				2	24	0	0	0 (0)	0	0	0 (0)
					3	19	0	0	0 (0)	0	0	0 (0)
					4	23	0	0	0 (0)	0	0	0 (0)
		PE-wt	NGG	NGG	1	24	0	9	9 (37.5)	5	4	9 (37.5)
					2	18	0	18	18 (100)	12	4	16 (88.9)
					3	16	2	14	16 (100)	6	8	14 (87.5)
					4	24	0	17	17 (70.8)	2	13	15 (62.5)
OsSSI2	A256T	PE-NG	NGc	NGt	1	24	1	0	1 (4.2)	0	0	0 (0)
		PE-wt	NGG	NGG	1	7	7	0	7 (100)	0	0	0 (0)
					2	24	7	0	7 (29.2)	0	2	2 (8.3)
					3	24	3	0	3 (12.5)	0	0	0 (0)
					4	24	6	0	6 (25.0)	0	0	0 (0)
					5	24	6	0	6 (25.0)	0	0	0 (0)
OsPDS	A31_stop	PE-NG	NGt	NGa	1	24	9	0	9 (37.5)	0	0	0 (0)
	(G to T and -C)				2	24	6	0	6 (25.0)	0	0	0 (0)
					3	24	0	0	0 (0)	0	0	0 (0)
					4	24	0	0	0 (0)	0	0	0 (0)
		PE-wt	NGG	NGG	1	24	24	0	24 (100)	0	0	0 (0)
					2	24	13	0	13 (54.2)	0	0	0 (0)
					3	24	2	0	2 (8.3)	0	0	0 (0)
					4	24	2	0	2 (8.3)	0	0	0 (0)

### 3.3 The PE-induced P177S mutations in the *OsEPSPS* gene confer herbicide resistance in rice

Although we attempted to obtain T_1_ progenies from T_0_ regenerated plants harboring homozygous *OsEPSPS-TIPS* gene to test glyphosate sensitivity, no T_1_ progenies were obtained. Thus, T_1_ progenies with homozygous P177S mutation in the *OsEPSPS* gene (*OsEPSPS-PS*) were obtained from regenerated plants with heterozygous *OsEPSPS-PS*, and their T_2_ progenies were subjected to glyphosate sensitivity testing (Supplementary Figure S3A). These plants showed a herbicide glyphosate-tolerant phenotype compared with wild-type plants (Supplementary Figure S3B). Furthermore, PCR analysis confirmed the presence of progeny plants carrying a homozygous *OsEPSPS-PS* gene and segregating the PE vector (Supplementary Figure S3C).

EPSPS catalyzes the transfer of the enolpyruvyl moiety of phosphoenolpyruvate to the 5-hydroxy position of shikimate-3-phosphate in the sikimate pathway, which is an essential pathway for the synthesis of aromatic amino acids (Funke et al., 2009). It has been reported that the *EPSPS-TIPS* mutant enzyme from *Escherichia coli* was essentially insensitive to glyphosate, while its activity was decreased significantly compared with wild-type enzyme (Funke et al., 2009). Thus, consistent with a previous report (Li et al., 2016), *OsEPSPS-TIPS* homozygous mutants were thought to be lethal.

## 4 Discussion

In this study, to explore which target sites PE-NG or PE-wt with paired epegRNAs recognize and modify efficiently, we compared PE efficiency using PE-NG with an nSpCas9-NG variant targeting NG-PAM and PE-wt with a canonical nSpCas9 targeting NGG-PAM. epegRNA-NG1s and -NG2s targeting *OsEPSPS*, *OsDL*, and *OsSSI2* gene recognized NGC- and NGT-PAM sites, respectively (Figure 1A; Supplementary Figure S1A,B). The NGT- and NGC-PAM sites were recognized by epegRNA-NG1 and -NG2 for the introduction of F140H mutations into the *OsHSL1* gene, respectively (Figure 1B), whereas paired epegRNAs targeting NGT- and NGA-PAM were designed to introduce an A31 stop mutation in *OsPDS* gene (Supplementary Figure S1C). Analysis of transgenic calli revealed that the PE efficiency of PE-wt with paired epegRNAs targeting NGG-PAMs was higher than that of PE-NG with paired epegRNAs targeting at least one NGC-PAM of two NG-PAMs even when NGG-PAMs were located distal to the target site (Figures 1, 2). On the other hand, the proportion of regenerated plants carrying the desired mutation derived from callus lines expressing PE-wt was consistently higher than that of regenerated plants expressing PE-NG at all target loci (Figure 3A; Table 1).

We first analyzed PE frequency in transgenic calli, calculated as the ratio of callus lines in which the desired mutation was detected to all transgenic lines analyzed. Thus, the proportion of the cells harboring the desired mutation in PE-positive callus was not considered in this step. The ratio of regenerated plants detected the desired mutation is thought to be proportional to the proportion of the cells carrying the desired mutation in PE-positive callus. This discrepancy in the PE efficiency via PE-NG and PE-wt at the *OsPDS* gene between transgenic calli and regenerated plants may be explained by the difference of the calculation methods.

To expand the targeting range, an SpCas9-NG variant recognizing NG PAMs has been developed by engineering based on the crystal structure of SpCas9 (Nishimasu et al., 2018). Although this variant can recognize NGH (where H is A, C, or T) PAM sites in addition to NGG-PAM, and induce DNA double-strand breaks, its cleavage activity was lower than that of wild-type SpCas9 at NGG sites, being particularly less active at NGC PAM sites relative to NGD (where D is G, A, or T) PAM sites in human cells (Nishimasu et al., 2018). SpCas9-NG (Nishimasu et al., 2018), xCas9 (Hu et al., 2018) and a near PAM-less SpCas9 (SpRY) (Walton et al., 2020) variants have been applied to PE to expand the range of target sites in mammalian cells (Kweon et al., 2021). However, the PE activities of PE2 with these SpCas9 variants was <10% at most target sites in HEK293T cells (Kweon et al., 2021). Thus, the PAM preferences of PE-NG might be one reason why PE-NG efficiency was low, especially at NGC-PAM, in this study. We expect that the PE efficiency at non-NGG PAM target sites could be improved by using other CRISPR/Cas members having different PAM preferences, such as SpRY with 5′-NR/YN-3′ PAM (Walton et al., 2020) and SaCas9 with 5′-NNGRRT-3′ PAM (Ran et al., 2015).

In the case of *OsHSL1*, *OsDL*, and *SSI2* modifications, undesired scaffold-derived byproducts were detected in more lines transformed with PE-wt compared with lines transformed with PE-NG (Supplementary Table S1). We have previously reported that improved PE efficiency led to a concomitant increase in the frequency of undesired scaffold-derived byproducts using paired epegRNAs in rice plants (Nishizawa-Yokoi et al., submitted). Accordingly, the incidence of undesired scaffold-byproducts was increased as PE efficiency improved in various previous studies, especially in plant cells (Lin et al., 2021; Zong et al., 2022). Therefore, we developed a high-fidelity epegRNA (epegRNA-HF) designed by modifying the scaffold sequence to eliminate scaffold-derived byproducts (Shuto et al., 2024) and applied it to rice genome editing (Nishizawa-Yokoi et al., submitted). The introduction of undesired scaffold-byproducts could largely be avoided by using an epegRNA-HF in rice.

## 5 Conclusion

PE with paired epegRNAs is a powerful approach with which to modify target genes efficiently and reproducibly, at least in rice. To expand the universality of PE with paired epegRNAs in rice, we compared PE efficiency of PE-wt with paired epegRNAs targeting the distal NGG-PAM *versus* PE-NG with paired epegRNAs targeting NG-PAMs adjacent to the target site. The PE frequency using PE-wt with paired epegRNAs targeting the distal NGG-PAM was higher than that of PE-NG with paired epegRNAs targeting NG-PAMs adjacent to the target site, at least when either paired epegRNA for PE-NG was designed to recognize NGC-PAM. These results might be due to PAM preferences of SpCas9-NG. Our findings suggest that paired epegRNAs should be designed to target NGG-PAMs for PE-wt as a first choice. If this is impossible, paired epegRNAs should be designed to target NGD-PAMs for PE-NG as a second choice to achieve precise and efficient genome editing in rice.

## Data Availability

The original contributions presented in the study are included in the article/Supplementary Material, further inquiries can be directed to the corresponding author.

## Supplementary material

SUPPLEMENTARY FIGURE S1 Target site and target sequence to modify the *OsDL*, *OsSSI2*, and *OsPDS* gene via PE-NG or PEwt with paired epegRNAs **(A)** Schematic of the rice drooping leaf (*OsDL)* gene target site (i) and target sequence to convert C44 to stop codon (-GC) via PE-NG (ii) or PE-wt (iii). **(B)** Schematic of the suppressor of salicylate insensitivity 2 (*OsSSI2*) gene target site (i) and target sequence to introduce an A256T (tGc to tAc) mutation via PE-NG (ii) or PE-wt (iii). **(C)** Schematic of the phytoene desaturase (*OsPDS*) gene target site (i) and target sequence to convert A31 to stop codon (G to C and -C) via PE-NG (ii) or PE-wt (iii). The sequences shown below the target sequences reveal the 3′extension region of the epegRNAs. PBS and RT templates are highlighted in light blue and yellow, respectively. The protospacer sequences for pegRNAs are underlined. The positions of the target amino acid residues are boxed. Target nucleotides are shown in red.

SUPPLEMENTARY FIGURE S2 Undesired scaffold-derived mutations were detected in regenerated plants carrying a PE-modified *OsHSL1* gene. **(A)** Target sequence of *OsHSL1* gene to introduce an F140H (TTt to CAt) mutation via PE-wt. The sequences shown below the target sequences reveal the 3′extension region of pegRNAs. The protospacer sequences for pegRNAs are underlined. **(B)** Representative sequence chromatogram of PCR products spanning the target site in the *OsHSL1* gene from regenerated plants.

SUPPLEMENTARY FIGURE S3 Plants harboring a homozygous *OsEPSPS-PS* gene show a glyphosate-resistant phenotype. **(A)** Representative sequence chromatogram of PCR products spanning the TIPS mutation site in the *OsEPSPS* gene from plants harboring a homozygous *OsEPSPS-PS* gene. The positions of the T173I and P177S mutations are boxed. **(B)** Glyphosate-resistant phenotype of wild-type (left) and T_2_ plants harboring homozygous *OsEPSPS-PS* gene (right). The seeds were sown on 1/2MS medium containing 0 (left panel), 25 (middle panel), and 50 (right panel) μM Glyphosate and were grown for 1-week before being photographed.
