# Supplementary material for: Prime editing using paired pegRNAs targeting NG- or NGG-PAM in rice
Source: Front Genome Ed. 2025 Aug 28;7:1550308. doi: 10.3389/fgeed.2025.1550308 (PMC12426525; doi:10.3389/fgeed.2025.1550308)

Supplementary Figure 1

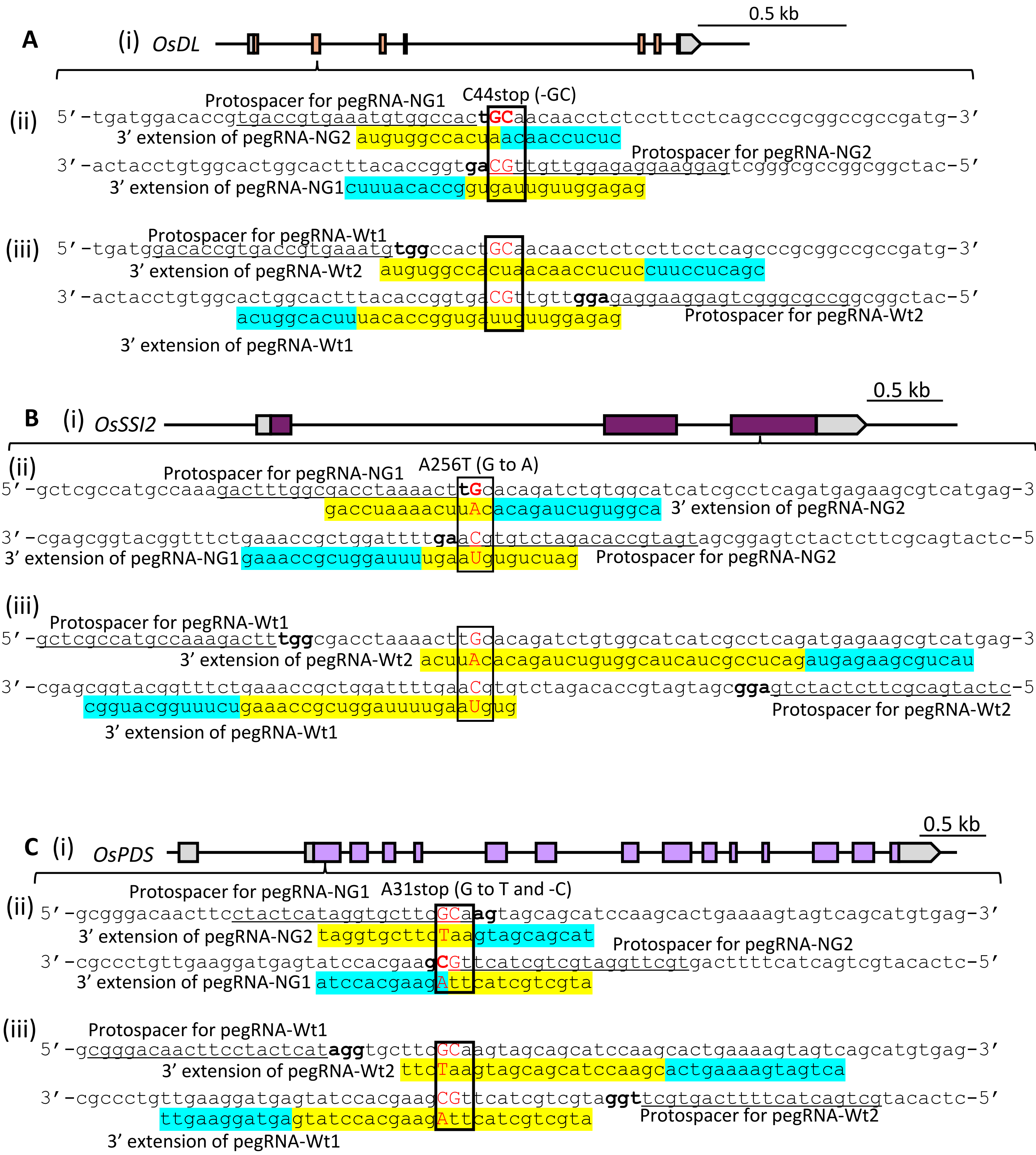

Supplementary Figure 2

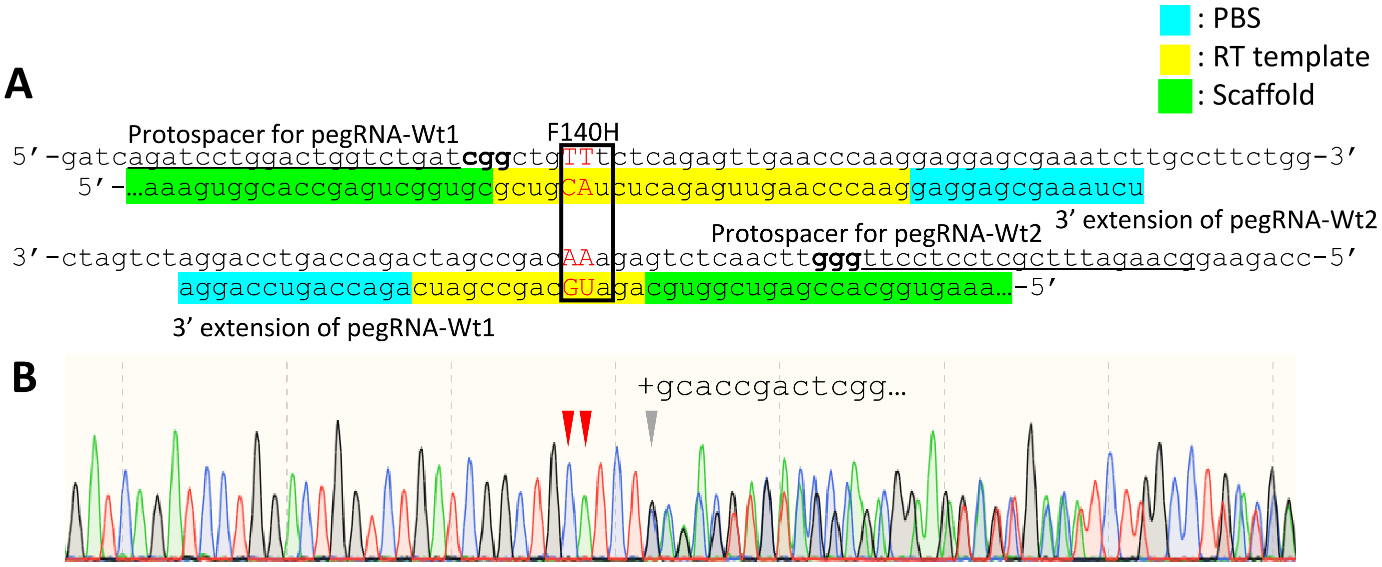

Supplementary Figure 3

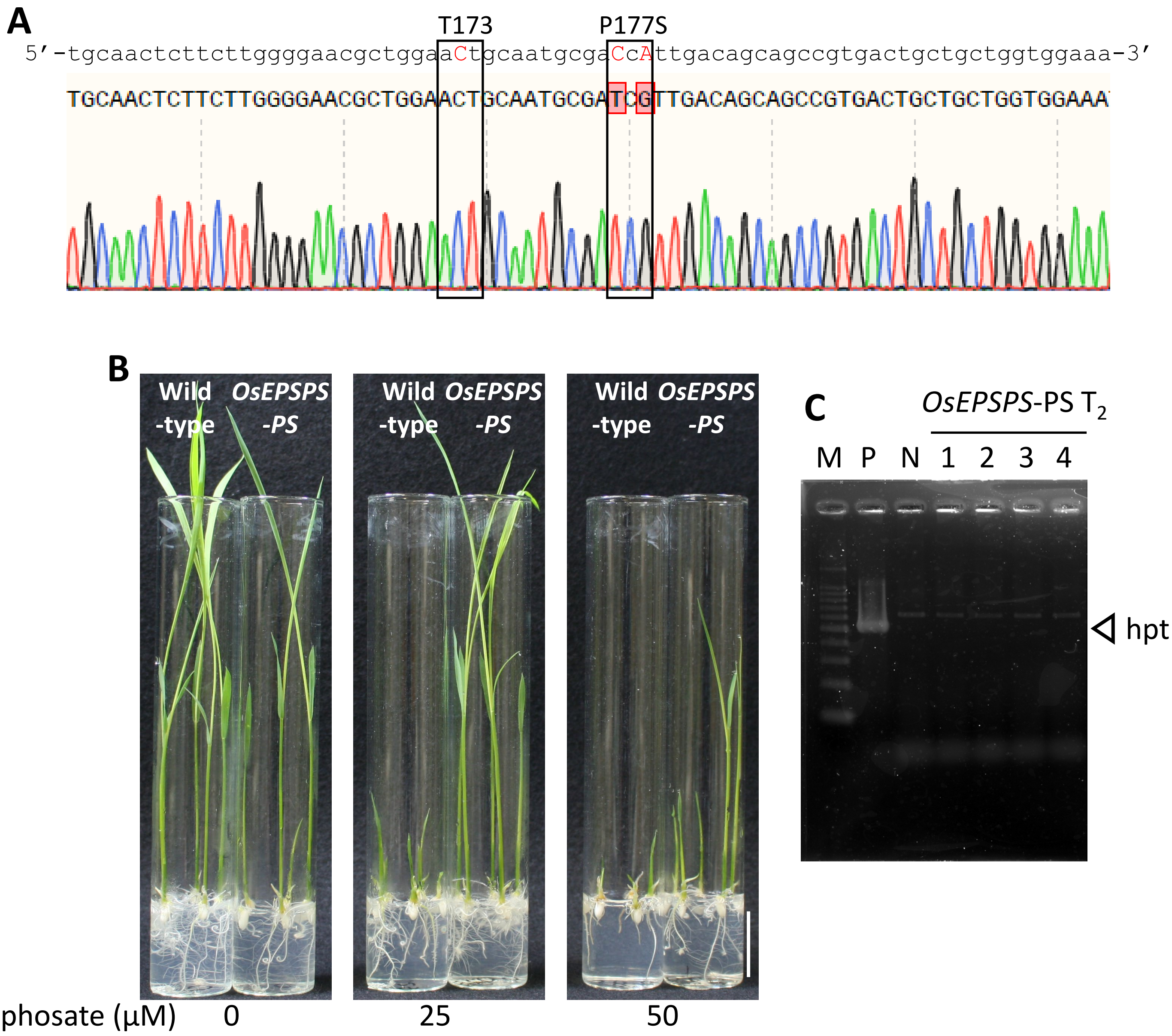

Supplement: Supplementary file 1 [file Supplementaryfile1.pdf]
